# Risk factors for severe dysmenorrhea in Arab women: A focus on war displacement and mental health outcomes

**DOI:** 10.3934/publichealth.2024010

**Published:** 2024-02-26

**Authors:** Omar Gammoh, Osama Abo Al Rob, Abdelrahim Alqudah, Ahmed Al-Smadi, Mohamad Obada Dobain, Reham Zeghoul, Alaa A. A. Aljabali, Mervat Alsous

**Affiliations:** 1 Department of Clinical Pharmacy and Pharmacy Practice, Faculty of Pharmacy, Yarmouk University, Irbid, 21163, Jordan; 2 Department of Clinical Pharmacy and Pharmacy Practice, Faculty of Pharmaceutical Sciences, The Hashemite University, Zarqa 13133, Jordan; 3 Adult Health Nursing, Prince Salma College, Al Al-Bayt University, Mafraq, Jordan; 4 Souriyat Across Borders, Amman, Jordan; 5 Department of Pharmaceutics and Pharmaceutical Technology, Faculty of Pharmacy, Yarmouk University, Irbid 21163, Jordan

**Keywords:** women, dysmenorrhea, war displacement, depression, anxiety, and insomnia

## Abstract

**Background:**

Dysmenorrhea is wide spread gynecological disorder among that affect the quality of life of women world wide. The current study aims to examine whether war displacement, mental health symptoms, and other clinical factors are associated with dysmenorrhea severity.

**Methods:**

This is a cross-sectional case-control study recruiting two groups: displaced Syrian women and un-displaced local Jordanian women. Demographics and clinical details were recorded. The severity of dysmenorrhea was assessed using WaLIDD scale, the PHQ-9 scale was emplyed to assess depressive symptoms, anxiety was assessed using the GAD-7 scale, and insomnia was assessed using the ISI-A scale. Predictors of severe dysmenorrhea in females using multivariate binary logistic regression.

**Results:**

Out of 808 of the total participants, 396 (49%) were Syrian displaced war refugees, 424 (42.5%) reported using paracetamol, 232 (23.2%) were using NSAIDs, and 257 (25.9%) using herbal remedies. Severe dysmenorrhea was associated with war displacement (*OR* = 2.14, 95% *CI* = 1.49–3.08, *p* < 0.001), not using NSAIDs (*OR* = 2.75, 95% *CI* = 1.91–3.95, *p* < 0.001), not using herbal remedies (*OR* = 2.01, 95% *CI* = 1.13–3.60, *p* = 0.01), depression (*OR* = 2.14, 95% *CI* = 1.40–3.29, *p* < 0.001), and insomnia (*OR* = 1.66, 95% *CI* = 1.14–2.42, *p* = 0.009).

**Conclusions:**

War displacement, type of analgesic, depression, and insomnia are risk factors for severe dysmenorrhea.

## 1. Introduction

Dysmenorrhea, painful menstruation, is a highly prevailing gynecological disorder that affects females, this condition is featured by painful menstrual cramps with a prevalence ranging from 30% to 94% [1],[2]. Ampile body of evidence describes the impact of dysmenorrhea on women's quality of life and daily functioning [3]. Dysmenorrhea severity has been shown to affect the social, academic, professional, and psychological well-being of women [4]–[6]. The relationship between dysmenorrhea and psychiatric disturbances is bi-directional.

Females with severe dysmenorrhea symptoms, according to the literature are more likely to experience psychiatic distress more than their peers with less symptoms severity. These psycgiatric symptoms include as anxiety, depression, and insomnia [7]–[11]. On the other hand, traumatized females with poor mental health are more likely to report severe dysmenorrhea [12]. War displacement is a major determinant of poor mental health, especially among females. Jordan is currently hosting about 1 million Syrian refugees compared with the last decade. Refugees have a greater risk for physical and psychological disorders such as anxiety and depression compared to the un-displaced population [13],[14].

Dysmenorrhea symptoms are managed by over-the-counter analgesics that include paracetamol (acetaminophen), non-steroidal anti-inflammatory drugs such as ibuprofen, and traditional herbal remedies [15]–[17]. Nonsteroidal anti-inflammatory drugs such as ibuprofen, diclofenac, and naproxen are considered the first-line treatment for dysmenorrhea [18], as they exert their analgesic and anti-inflammatory effect by inhibiting cyclooxygenase (COX) enzymes, including COX-1 and COX-2 [19],[20]. Despite the availability of various NSAIDs, none were found to be superior, as all were shown to be superior to placebo in relieving dysmenorrhea pain and improving quality of life [21]. Complementary medicine and herbal therapy are used for dysmenorrhea[15]; examples include fennel, chamomile, and thyme [22]. These herbs were found to improve dysmenorrhea symptoms by exerting anti-inflammatory, antispasmodic effects [23]. Understanding the determinants of severe dysmenorrhea is essential for its proper management and subsequently to improve women's quality of life. According to our knowledge, the effects of war displacement, dysmenorrhea analgesic type, psychiatric symptoms, and dysmenorrhea severity were rarely studied [24]. Therefore, we aim to examine the association between war displacement, mental health symptoms, and dysmenorrhea severity in a cohort of women in Jordan.

## 2. Materials and methods

### 2.1. Study design and settings

A cross-sectional study design was employed to achieve the study objectives. This study protocol has been approved by Yarmouk University IRB committee under the number (39/2022), additionally, the managagment of Caritas-Jordan (a non profit organization that stems from the Catholic Church) approved the study and provided access to the database of their beneficiaries

### 2.2. Participant recruitment

After obtaining the data set for female Syrians, the study sample was approached using individual phone calls to explain the study objective and protocol. Then, to maximise the privacy of the respondents, the link containing the study questionnaire was sent to the willing females where the first step was to sign the consent form electronically before the enrollement in the study. In regards to the Jordanian sample, a convenient sampling method was used for recruitment through different female-dedicated social media platforms. To reduce data bias, participants reporting menopause or who had received a gynecological surgery such as a hysterectomy or radiation therapy were all excluded. The study sample size for refugees and Jordaninas was informed based on sample size caculations on a confidence level of 95%, a confidence interval of 5%, and an estimated population size of 1 million refugees.

### 2.3. Data collection and study instrument

Data were collected electronically via a link sent to the potential participants. The link directs the participants to the study instrument that was uploaded on a Google form. All the participants were first asked for their willingness to participate by choosing “ I agree” to participate. The research team assisted participants with low literacy (from refugees) by reading out the study questionnaire over the phone.

#### 2.3.1. Covariates

A well-designed self-administered questionnaire was employed to obtain relevant demographic and clinical information of the study sample. These included information regarding the participants' age, height, weight, highest education achieved, current employment status, and previous diagnosis with chronic diseases.

#### 2.3.2. Exposure

The information about the self-medication with analgesics of the study sample was collected. Participants were asked to choose one or more options from a checklist presenting all the over-the-counter analgesics. In addition, to improve the quality of the collected data, the generic name, the brand name, and the picture of the medication pack were all presented and checked out by the patients.

The self-medication list comprised: Acetaminophen, Non-Steroidal Anti-inflammatory Drugs (NSAIDs) such as (Ibuprofen, Diclofenac, and Naproxen), muscle relaxants, herbal medicine, vitamins, sports, and no medication. The next section was dedicated to estimating the dosing frequencies of the selected analgesics as per the following choices: more than one dose per day, only one dose per day, on intermitted days, and none at all.

The Patient Health Questionnaire-9 (PHQ-9) Arabic-validated version was used to assess the severity of depressive symptoms. This self-administered scale stems from the Diagnostic and Statistical Manual of Mental Disorders-IV criteria for diagnosing depression [25], has a sensitivity of 88%, specificity of 88%, and reliability of (Chronbach's alpha = .88) for depression, and is widely used in Arabic speaking subjects [26], a cut-off score exceeding 14 is indicative for severe depressive symptoms [27].

The General Anxiety Disorder-7 (GAD-7) Arabic-validated scale was employed to assess the severity of anxiety symptoms, the scale indicates severe anxiety symptoms at a score of 15 and higher [28]. The scale showed excellent reliability (Chronbach's alpha = 0.95) among Arab-speaking subjects [26].

The assessment of the insomnia severity was carried out using the Insomnia Severity Index-Arabic version (ISI-A). This self-administered scale developed by Morin et al,[29] was translated and validated and showed good reliability (Chronbach's alpha = 0.84) determining severe insomnia at a threshold score of 15 [30],[31].

The Perceived Stress Scale-Arabic version (PSS-A) was used to provide an assessment of the severity of stress symptoms. The scale originally developed by Cohen and Williamson [32] comprises fourteen items designed to assess stress severity for the past month, a threshold score of fifteen or above indicates severe stress symptoms. We used the Arabic version of the scale that demonstrated reliability (Chronbach's alpha = .88) among Arab-speaking individuals [33].

#### 2.3.3. Outcome measurements

The severity of dysmenorrhea symptoms is the outcome variable of the study. This variable was assessed using WaLIDD (working ability, location, intensity, days of pain, dysmenorrhea) scale as in [34]. This self-administered scale provides a comprehensive insight into dysmenorrhea including the pain location(s), the pain severity, the number of painful days of menstruation, and the disability to carry out daily activities. The WaLIDD scale generates a score range between 0 to 12. A threshold score of 8 or higher is indicative of severe dysmenorrhea. The Arabic version used demonstrated reliability (Cronbach's alpha = 0.72) [35].

### 2.4. Statistical analysis

Frequencies, percentages, means, and standard deviations were used to describe the sample sample features. To investigate the association between the different classes of analgesics and other covariates with the main outcome variable (dysmenorrhea severity) a chi-square univariate analysis was carried out. Potential confounders demonstrating *p* < 0.10 were then used to feed the backward step-wise multiple regression model with (dysmenorrhea severity) as the dependent variable. Statistical significance was set at 2-sided *p* < 0.05 and estimates were set at 95% *CI*.

## 3. Results

### 3.1. Demographic characteristics

**Figure 1.**
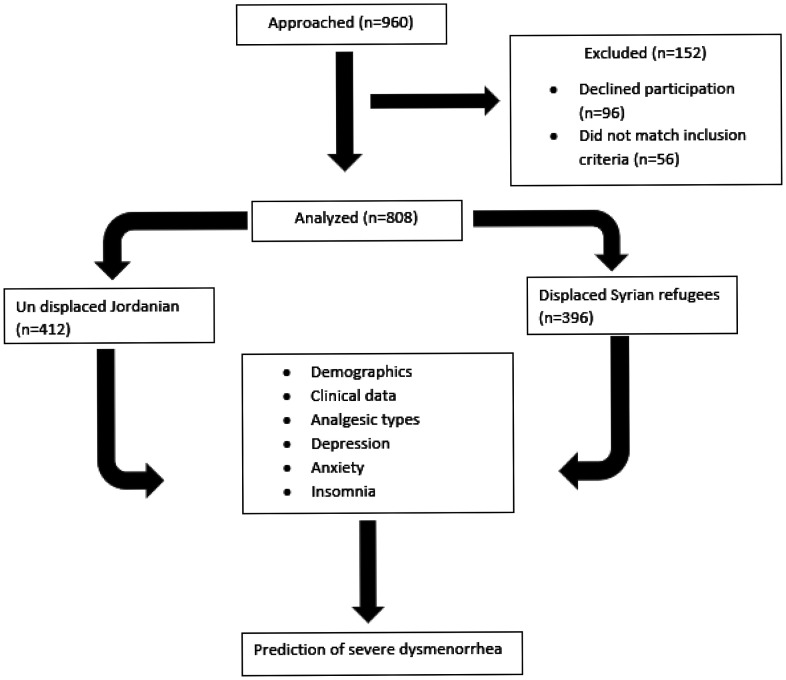
Study flow chart.

A total of 960 participants were approached, 96 declined participation and 56 were excluded because they did not fit in the inclusion criteria (Figure 1).

Out of 808 total participants analyzed, 396 (49 %) were Syrian female refugees and 412 (51%) were Jordanian females. The mean age was 31.82 ± 9.49 years. About half of the participants had a university level of education. Most of the Syrian refugees had no jobs (87.63) and were married (80.81). Data are presented in Table 1.

### 3.2. Working ability, location, intensity, days of pain, and dysmenorrhea (WaLLLID)

The WaLLID score was measured using 4 questions related to working ability, pain location, intensity of pain, and duration of pain in days. The mean WaLLID score for Syrian refugee participants was 6.83 ± 2.43, which was significantly higher than the score of Jordanian participants (*p* < 0.01). About 48.76% of participants reported that they had pain in two to three sites during dysmenorrhea. Only 15.10% of participants had dysmenorrhea pain that lasted for more than 5 days.

**Table 1.** Respondent's demographic characteristics.

**Project**	**Number**	**Syrian (*n* = 396)**	**Jordanian (*n* = 412)**
**• Age (Mean ± SD)**	31.82 ± 9.49	30.51 ± 9.80	33.07 ± 9.02
**• Height (Mean ± SD)**	164.24 ± 69.10	163.18 ± 65.92	165.26 ± 72.08
**• Weight (Mean ± SD)**	68.65 ± 16.26	68.88 ± 17.94	68.44 ± 14.48

**Level of Education**			
**• Below University level**	394 (43.2)	331 (83.6)	63 (15.3)
**• University level**	414 (51.24)	65 (16.4)	349 (84.7)

**Have Chronic diseases**			
**• Yes**	173 (21.4)	103 (26.0)	70 (17.0)
**• No**	635 (78.6)	293 (74.0)	342 (83.0)

**Career**			
**• Does not work**	272 (33.7)	347 (87.6)	151 (36.7)
**• Work**	498 (61.6)	38 (9.6)	234 (56.8)
**• Student**	38 (4.7)	11 (2.8)	27 (6.6)

**Marital status**			
**• Single**	234 (29.0)	30 (7.6)	204 (49.5)
**• Married**	506 (62.5)	320 (80.8)	186 (45.1)
**• Divorced**	44 (5.5)	31 (7.8)	13 (3.2)
**• Widowed**	24 (3.0)	15 (3.8)	9 (2.2)

**Place of living**			
**• Amman**	381 (47.2)	138 (34.8)	243 (59.0)
**• Zarqa and Mafraq**	137 (17.0)	107 (27.0)	30 (7.3)
**• North Jordan**	226 (28.8)	121 (30.6)	105 (25.5)
**• South Jordan**	12 (1.5)	1 (0.3)	11 (2.7)
**• Balqaa**	27 (3.3)	14 (3.5)	13 (3.2)
**• Madaba**	8 (0.1)	4 (1.0)	4 (1.0)
**• Jerash**	17 (2.1)	11 (2.8)	6 (1.5)

**Smoking**			
**• Not smoker**	637 (78.8)	363 (91.7)	274 (66.5)
**• Smoker**	141 (17.5)	19 (4.8)	122 (29.6)
**• Quit smoking**	30 (3.7)	14 (3.5)	16 (3.9)

**Menses pattern**			
**• Irregular menses**	127 (15.7)	80 (20.2)	47 (11.4)
**• Regular menses**	597 (73.9)	264 (66.7)	333 (80.8)

**Type of analgesic used**			
**• Paracetamol**	424 (42.5)	238 (42.6)	186 (42.4)
**• NSAIDs**	232 (23.2)	88 (15.8)	144 (32.8)
**• Herbal**	257 (25.9)	188 (33.6)	69 (15.8)
**• No analgesics**	84 (8.4)	45 (8)	39 (9.0)

### 3.3. Depression

The mean score of the Patient Health Questionnaire (PHQ-9) was 14.68 ± 6.41, which indicates moderate-severe depression among respondents.

When participants were asked about their interest in doing things, 21.63% of them had a high desire to do their activities. More than 40.0% stated that they were very depressed and feeling hopeless and they felt very bad about themselves, while 15% stated that they had thoughts of being better off dead.

### 3.4. Anxiety

According to the (GAD-7) scale, about a third of the participants reported that they have no control over worrying and distressing too much nearly every day. The mean GAD-7 score was 12.16 ± 6.20, where more than a third of the participants were categorized as anxious. There was no significant difference between the mean (GAD-7) scores of Syrian and Jordanian participants, with *p* = 0.563.

### 3.5. Insomnia

The ISI scale was used to rate the nature and symptoms of insomnia. About 15% of participants stated that they had very severe difficulty in falling or staying asleep and they worry about their current sleep problem. The mean ISI score was 13.19 ± 7.71, where more than half of the participants were categorized as having no insomnia. There was no significant difference between the mean (ISI) scores of Syrian and Jordanian participants, with *p* = 0.741.

### 3.6. Factors affecting severe dysmenorrhea

The association of socioeconomic factors with severe dysmenorrhea (≥8 score of WaLLID scale) was assessed using Chi-square analysis, and there was no significant association between severe dysmenorrhea and occupation or marital status and smoking (*p* > 0.05); however, young age, war displacement, an educational level less than university level, not having a job, having chronic diseases, having irregular menses, not taking NSAIDs or herbals, having anxiety, depression, and insomnia were significantly associated with severe dysmenorrhea (*p* < 0.05). (Table 2).

Significant factors were subjected to binary logistic regression analysis (backward LR) and results showed that young age, war displacement (Syrian refugees), screened for severe depression, screened for severe anxiety, screened for severe insomnia, not taking NSAIDs, and not taking herbal preparations were predictors of severe dysmenorrhea in females. (Table 3).

**Table 2.** Factors associated with severe dysmenorrhea (*n* = 808).

Variable	Severe dysmenorrhea/N (%)	No severe dysmenorrhea/N (%)	*p* value
Age (Years)			<0.001*
≤30	174	253	
>30	86	250	
Nationality			<0.001*
Jordanian	105	223	
Syrian	156	281	
Educational level			0.001*
Less than University level	150	226	
University level	111	278	
Occupation status			0.02*
Working	71	186	
Not working	178	292	
Student	12	26	
Having chronic diseases			0.029*
Yes	64	90	
No	197	414	
Smoking			0.141
Smoker	35	96	
Not smoker	215	390	
Quit smoking	11	18	
Marital status			0.154
Single	75	153	
Married	160	317	
Divorced	21	21	
Widow	5	13	
Menses pattern			0.016*
Irregular menses	54	67	
Regular menses	190	389	
Use of paracetamol as analgesic			0.038*
Yes	132	293	
No	131	212	
Use of NSAIDs as analgesic			<0.001*
Yes	114	119	
No	149	386	
Use of vitamins as analgesic			0.245
Yes	35	53	
No	228	452	
Use of herbals as analgesic			<0.001*
Yes	40	33	
No	223	472	
Having insomnia			<0.001*
Yes	158	175	
No	103	329	
Having anxiety			<0.001*
Yes	147	153	
NO	111	328	
Having depression			<0.001*
Yes	183	207	
No	77	290	

Note: *p < 0.05.

**Table 3.** Predictors of severe dysmenorrhea in females using binary logistic regression.

**Independent variable**	**B**	**SE**	**Odds ratio**	**95% *CI***	***p*-value**
Having depression	0.76	0.22	2.14	1.40–3.29	0.001*
Having anxiety	0.39	0.21	1.47	0.98–2.21	0.060
Not taking NSAIDs	1.01	0.19	2.75	1.91–3.95	<0.001*
Not taking herbals	0.70	0.29	2.01	1.13–3.60	0.017*
Having insomnia	0.51	0.19	1.66	1.14–2.42	0.009*
Age ≤30 years	-0.57	0.18	0.57	0.40–0.80	0.001*
War displacement	0.76	0.19	2.14	1.49–3.08	<0.001*

Note: *p < 0.05.

## 4. Discussion

We aimed to explore the associations between war displacement, analgesics, psychiatric symptoms, and dysmenorrhea severity. War displacement, not taking NSAIDs or herbals, depression, and insomnia were all significantly associated with severe dysmenorrhea. The findings make an important contribution to the existing literature on women's health in the field of war displacement and dysmenorrhea.

Displaced women, due to warfare, who are reporting mental health disturbances were shown to be at higher risk of experiencing severe dysmenorrhea compared to undisplaced women [36]. Chronic pain, according to large-scale cross-sectional studies, is a very common symptom that prevails in 20% of women according to the literature [37]. Furthermore, the pain-related disability and functional impairment are related to depression and lower productivity, which can be 5 times greater compared to subjects with no pain [39]–[41]. Previous studies carried out ten years ago on samples of Syrian refugees residing in Jordan showed a high prevalence of depression, anxiety, and insomnia [41].

Our findings revealed that depression, anxiety, and insomnia were all associated with severe dysmenorrhea. This can be explained by alterations in the hormonal and neurotransmitters that could lead to being more sensitive to chronic pain [31]. The management of the mental health status of Syrian war veterans is challenging and complex due to the presence of post-migration stressors that play a substantial role in the individuals' quality of life, daily functioning, and health. These post-migration stressors involve poor living conditions, chronic diseases, chronic medications that could affect mood status, and many others[42]. Therefore, a holistic approach involving both non-pharmacological and pharmacological approaches may be effective such as heat therapy, psychotherapy, and massage [43].

Our result showed that women who did not use NSAIDs or herbs reported higher odds of severe dysmenorrhea. Although dysmenorrhea is the most common painful condition reported by females, we suggest that due to cultural reasons and stigma, females cannot explicitly express menstrual cycle-related problems in primary health care settings and are under-informed in regards to menstrual hygiene and are unprepared for menarche [44].

The inappropriate pharmacological treatment of dysmenorrhea pain is linked to severity. Dysmenorrhea pain is a consequence of an inflammatory cascade characterized by prostaglandin synthesis that is inhibited by the NSAIDs [45]. The use of NSAIDs is highly recommended in dysmenorrhea [21], with several randomized studies supporting this practice [46],[47]. Similarly, the efficacy of herbal remedies such as fennel, thyme, and anise is based on their anti-inflammatory action [15],[23]. The topics related to menstrual cycle practices are under-represented in the literature and are poorly practiced in developing countries' communities, especially among refugees where access to private clean sanitary facilities could be difficult [44]. This is the first study that examines war displacement, analgesic type, and psychiatric symptoms as predictors for severe dysmenorrhea. The study strengths include the factors studied, the sample type, and the sample size. On the other hand, the cross-sectional design and the self-assessment tools are considered limitations of the study.

## 5. Conclusions

In conclusion, war displacement, the type of analgesic, and mental health symptoms are associated with severe dysmenorrhea in Arab women living in developing countries. Close monitoring of the mental health status and pharmacotherapy is required to optimize women's health. More awareness campaigns, support groups, and individual counseling about menstrual pain and its management by healthcare providers are required to optimize women's health.

## Use of AI tools declaration

The author declares no Artificial Intelligence (AI) tools have been used in the creation of this article.
